# Postoperative Radiation Therapy for Parotid Mucoepidermoid Carcinoma

**DOI:** 10.1155/2014/345128

**Published:** 2014-12-14

**Authors:** Meghan P. Olsen, Allen O. Mitchell, Edward F. Miles

**Affiliations:** ^1^Uniformed Services University, 4301 Jones Bridge Road, Bethesda, MD 20814, USA; ^2^Division of Otolaryngology, Department of Surgery, Naval Medical Center Portsmouth, 620 John Paul Jones Circle, Portsmouth, VA 23708, USA; ^3^Division of Radiation Oncology, Department of Radiology, Naval Medical Center Portsmouth, 620 John Paul Jones Circle, Portsmouth, VA 23708, USA

## Abstract

Salivary gland cancers are rare and represent approximately 5% of all head and neck cancers and only 0.3% of all malignancies. The majority (75%) of salivary gland tumors occur in the parotid gland, and while benign lesions are more common, mucoepidermoid carcinoma (MEC) makes up 40–50% of malignant parotid gland tumors. No randomized controlled trials exist regarding the role of adjuvant radiation for patients who undergo surgical resection of low-grade MECs. Herein, we report two cases of successful postoperative radiation therapy in low-grade, pT2N0 MEC of the parotid gland. The role of adjuvant radiation therapy for patients with MEC of the parotid gland is based on data from institution reviews and lacks data from randomized controlled trials. Per our review of the literature, the pathological findings of positive surgical margins and/or perineural invasion in two patients with low-grade MEC of the parotid gland warranted adjuvant radiation for improved local control after partial parotidectomy. Both patients tolerated postoperative radiation therapy with only mild side effects and, at last follow-up, five years after completion of therapy, had no clinical or radiographic evidence of either local recurrence or distant metastasis.

## 1. Introduction

Salivary gland cancers are rare and represent approximately 5% of all head and neck cancers and only 0.3% of all malignancies [1]. The majority (75%) of salivary gland tumors occur in the parotid gland, and while benign lesions are more common, mucoepidermoid carcinoma (MEC) makes up 40–50% of malignant parotid gland tumors [1–3]. MECs were first described by Stewart et al. [4] in 1945 and are made up of three cell types in differing proportions: mucous-producing cells, epidermoid cells with squamous differentiation, and undifferentiated small cells. These tumors are typically classified into three histologic grades (low, intermediate, or high) according to the Armed Forces Institute of Pathology (AFIP) grading scheme, based on characteristics such as presence of intracystic components, neural invasion, necrosis, anaplasia, and level of mitotic activity. The histologic grading of these lesions has been shown to strongly correlate with clinical behavior and overall prognosis [2, 5].

Patients with salivary gland tumors commonly present with a mass and/or localized pain. Management of these cancers typically involves surgical resection with postoperative radiation therapy considered in cases where there is concern for local failure based on pathological assessment. Though previously believed to be radioresistant, generally accepted indications for adjuvant radiotherapy in MEC include positive surgical margins, positive nodal involvement, extracapsular extension, perineural invasion, and advanced stage (T3 and T4) [6]. While a previous randomized clinical trial was performed examining the role of radiation therapy for unresectable salivary gland tumors, no randomized controlled trials exist regarding the role of adjuvant radiation for patients who undergo surgical resection [7].

Herein, we report two cases of successful postoperative radiation therapy in low-grade, pT2N0 MEC of the parotid gland.

## 2. Case 1

The patient is a 27-year-old African American male who presented in October 2008 with a 5-year history of a mass in the area beneath his left ear. It was originally noted to be approximately 1 cm in size but, by the time of presentation, it had grown to almost 2 cm and had become painful. He sought medical attention and a trial of antibiotics was unsuccessful in resolving the mass. A CT of the maxillofacial area in late October 2008 revealed a subtle, mixed isodense nonenhancing mass measuring 2 × 1.6 cm in the left parotid gland with no adenopathy in the neck. MRI in December 2008 again demonstrated a 1.3 cm mass in the superficial aspect of the left parotid gland without adenopathy.

Fine needle aspiration (FNA) of the lesion on December 23, 2008, returned inflammatory cells consistent with sialadenitis but could not rule out a low-grade malignancy. Resection of the lesion via a nerve sparing left parotidectomy in January 2009 demonstrated a 2.7 cm low-grade MEC with involved margins and perineural invasion. This tumor was classified as low grade according to the AFIP grading scheme, with a total score of 2/14 (intracystic component < 20%: 0/2; presence of neural invasion: 2/2; necrosis: 0/3; mitoses: 0/3; anaplasia: 0/4). There was no evidence of lymphovascular invasion and four lymph nodes were excised and pathologically negative for carcinoma.

On physical exam two weeks after surgery, the patient's House-Brackmann score was rated II/VI. The patient exhibited complete eye closure with maximal effort, symmetric facies at rest, and mild weakness of the marginal mandibular and buccal branches. Four months later, his House-Brackmann score improved to I/VI, though he continued to complain of xerostomia on the left side of his mouth, left side face and neck pain, some discomfort with swallowing, and a left sided headache. He was referred to Radiation Oncology for additional evaluation and management.

At his initial consultation appointment in February 2009, physical exam revealed a healing incision in the left auricular area consistent with his recent parotidectomy with no lymphadenopathy. After a careful review of the available literature regarding this rare malignancy, he was offered adjuvant radiation therapy to the left parotid tumor bed based on the findings of positive surgical margins and perineural invasion.

The radiation fields included the existing tumor bed with appropriate margin to cover microscopic disease spread, patient motion, and daily set-up error, with daily 200 centigray (cGy) fractions to a total dose of 6,000 cGy. The risks, benefits, and alternatives to adjuvant radiation therapy in this context were discussed in detail with the patient and informed consent was obtained.

Intensity modulated radiation therapy (IMRT) was delivered via a TomoTherapy machine (TomoTherapy, Madison, WI), with a 6 MV photon beam. He completed treatment as planned with no breaks required and experienced only mild xerostomia, dysgeusia, and hyperpigmentation in the skin of the treatment field. His physical exam and MR imaging 5 years after completion of therapy demonstrated no evidence of recurrent disease.

## 3. Case 2

This patient is a 30-year-old Caucasian male who presented with a 2-year history of a 2 cm, nontender mass below his right ear. FNA of the lesion on December 4, 2007, demonstrated a chronic lymphocytic infiltrate consistent with a Warthin's tumor versus sialadenitis. At this time, resection of the mass was recommended by Otolaryngology but the patient declined. An MRI of the neck on January 29, 2009, demonstrated a 2.9 × 1.6 cm heterogenous mass in the right deep parotid with multiple other smaller lesions adjacent to it without lymphadenopathy. He underwent nerve sparing parotidectomy with a sternocleidomastoid flap on February 3, 2009. Pathology demonstrated a 2 cm low-grade MEC in the deep lobe of the parotid with a positive surgical margin. This tumor was also classified as low grade according to the AFIP grading scheme, with a total score of 2/14 (intracystic component > 20%: 2/2; presence of neural invasion: 0/2; necrosis: 0/3; mitoses: 0/3; anaplasia: 0/4). Nine lymph nodes were excised and were pathologically negative for disease. There was no evidence of lymphovascular invasion.

On physical exam, six weeks after surgery, this patient's House-Brackmann score was rated IV/VI due to documented incomplete eye closure. Two months later, his House-Brackmann score improved to II/VI, exhibiting full eye closure and only mild smile asymmetry. By November 2009, the House-Brackmann score was I/VI, and the patient complained only of some areas of numbness in the buccal distribution of V2. He was referred to Radiation Oncology for additional evaluation and management.

Based on the finding of positive surgical margins, the patient was offered adjuvant radiation therapy. He was counseled and subsequently treated in the same fashion as the previous patient, with IMRT delivered via TomoTherapy machine (TomoTherapy, Madison, WI), with the same fractionation schedule and total dose. He had no significant difficulty during treatment, with only some mild xerostomia, dysgeusia, and hyperpigmentation in the skin of the treatment field. His physical exam and MR imaging 5 years after completion of therapy demonstrated no evidence of recurrent disease.

## 4. Discussion

The mainstay of treatment for salivary gland tumors, in general, and mucoepidermoid carcinomas, specifically, is surgical resection. For intermediate or high-grade MEC, adjuvant radiation therapy appears to aid in local control (if not overall survival) in cases of advanced staging, close or positive surgical margins, high-grade histology, perineural invasion, or lymphovascular invasion. In the case of low-grade disease, the indications are less clear; traditionally, low-grade disease has been treated with surgical excision alone [2]. In both of the above reported cases of low-grade MEC, the surgical margins were positive and one case also demonstrated perineural invasion. Although frozen section is often useful in intraoperative decision making, preoperative FNA consistent with low-grade malignancy established the decisions to spare the functioning facial nerve in these patients. Therefore, it was felt that frozen section would have limited utility in these cases, and the high likelihood of positive margin was accepted in order to preserve facial nerve function. For the same reasoning, reexcision was not considered [8]. Although there are no randomized trials to guide treatment recommendations in this particular setting, the literature is replete with retrospective reviews, mostly from single institutions.

In a 1997 study from Garden [9], comprising a retrospective review of 166 patients, Garden et al. recommended postoperative radiation therapy for patients with high-grade histology, recurrent disease, inadequate surgical margins, perineural invasion, nodal disease, or extension of the disease beyond the gland. Improved local control was achieved for doses greater than 60 Gy, with local recurrence occurring in only 2% of patients. Unfortunately, this review included all parotid tumors, only 28% of which were MECs [9]. More recently, Chen et al. reviewed 61 patients with localized MEC treated with surgery and postoperative radiation therapy to a median dose of 60 Gy. The 5-year estimate of overall survival for patients with non-high-grade tumors was 83% [10]. Terhaard et al. examined the role of radiation therapy in the treatment of malignant salivary gland tumors, and although surgery alone was generally recommended for MEC, postoperative radiation improved 10-year local control for patients with malignant tumors and advanced T stage (T3 and T4), close or positive surgical margins, bone invasion, or perineural invasion [11].

Indications for treatment of the neck, especially for patients with clinically negative lymph nodes (N0), remain unclear. The strongest predictors for occult lymph node metastasis in a clinically N0 patient have traditionally been tumor histology, pathologic grade, tumor size, and site of disease [12]. Armstrong et al. reported that patients with small low-grade tumors were considered to have minimal risk of occult nodal disease, making elective treatment of the neck unnecessary [13]. However, several strategies are currently accepted for management of the clinically N0 neck for patients with salivary gland carcinomas including elective neck dissection, close observation, and elective neck irradiation [12].

De Brito Santos et al. [14] recommended elective neck treatment for patients with parotid carcinoma and clinically N0 disease for high-risk histological types (i.e., adenocarcinoma, undifferentiated carcinoma, salivary duct carcinoma, squamous cell carcinoma, and high-grade MEC) and for those with advanced T stage (T3 or T4). They reported occult metastases in 22.2% of 145 patients with either of these features [14]. In a single institution retrospective review from 2008 covering 52 cases from Finland over a period of 30 years, no patient with low-grade disease was found to have positive lymph nodes at resection or during follow-up despite not having received radiation therapy [3]. Lau et al. performed a retrospective study of 119 patients with clinically N0 salivary gland carcinoma, 47% of which had MEC. Their findings found that the incidence of occult lymph node metastases was higher in high-grade MEC (35%) and adenocarcinoma (35%) whereas patients with low-grade MEC had low rates of nodal involvement, suggesting that elective neck irradiation would not likely benefit patients with low-grade disease [15].

In conclusion, the role of adjuvant radiation therapy for patients with mucoepidermoid carcinoma of the parotid gland is based on data from institution reviews and lacks data from randomized controlled trials. Per our review of the literature, the pathological findings of positive surgical margins and perineural invasion in two patients with low-grade MEC of the parotid gland warranted adjuvant radiation for improved local control after parotidectomy. For these reported patients with low-grade MEC, direct treatment of the neck was not performed.

Both patients tolerated postoperative radiation therapy with only mild side effects and, at last follow-up, five years after completion of therapy, had no clinical or radiographic evidence of either local recurrence or distant metastasis.
